# Enterovirus Control of Translation and RNA Granule Stress Responses

**DOI:** 10.3390/v8040093

**Published:** 2016-03-30

**Authors:** Richard E. Lloyd

**Affiliations:** Department of Molecular Virology and Microbiology, Baylor College of Medicine, Houston, TX 77030, USA; rlloyd@bcm.edu; Tel.: +1-713-798-8993

**Keywords:** enterovirus, poliovirus, coxsackievirus, translation shutoff, stress granules, P-bodies

## Abstract

Enteroviruses such as poliovirus (PV) and coxsackievirus B3 (CVB3) have evolved several parallel strategies to regulate cellular gene expression and stress responses to ensure efficient expression of the viral genome. Enteroviruses utilize their encoded proteinases to take over the cellular translation apparatus and direct ribosomes to viral mRNAs. In addition, viral proteinases are used to control and repress the two main types of cytoplasmic RNA granules, stress granules (SGs) and processing bodies (P-bodies, PBs), which are stress-responsive dynamic structures involved in repression of gene expression. This review discusses these processes and the current understanding of the underlying mechanisms with respect to enterovirus infections. In addition, the review discusses accumulating data suggesting linkage exists between RNA granule formation and innate immune sensing and activation.

## 1. Enteroviruses Rapidly Shut Down Host Translation

The enterovirus genome is a functional mRNA that must be expressed as the first step of the infectious cycle; thus it is of no surprise that enteroviruses exert rapid and dominant control over the cellular translation apparatus. Enteroviruses repress cellular translation, transcription and DNA replication during infection, but repression of translation occurs first and is the most drastic. Since the cellular translation apparatus quickly responds to stress and altered environmental conditions, it is important for the virus to strongly regulate multiple aspects of the translation apparatus to not only promote its own gene expression, but to restrict those cell responses that activate innate immunity and may limit access to metabolites and components needed by the virus.

Human enteroviruses such as poliovirus (PV) and coxsackievirus B3 (CVB3) express two viral proteinases that work together to rapidly shut down host cap-dependent translation. Like cellular mRNA, enterovirus RNA contains a poly(A) tail, but does not contain the 5′ m^7^GTP cap structure that is common to virtually all cellular mRNAs and serves the function of a translation promoter. Enteroviruses exploit this structural difference to shutoff cap-dependent translation machinery, and thus cap-dependent translation, while promoting cap-independent translation mechanisms that the virus uses.

**Figure 1:**
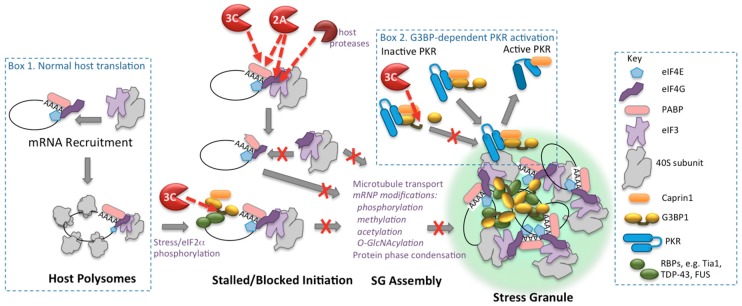
Cleavage-based mechanisms of enterovirus control of translation apparatus and stress granules. Box 1 shows the normal cap-dependent translation initiation mRNA binding step and mature polysomes. Red dashed arrows indicate substrate targets of viral proteinases. Box 2 depicts G3BP1-dependent activation of PKR through recruitment to stress granules (SGs), followed by its release. Only components discussed in the text are shown, many SG components are not depicted.

The mechanism of enterovirus shutoff of host translation is well worked out and principally results from cleavage of translation initiation factors, eIF4G and PABP. Within two to three hours of infection, poliovirus or CVB3 2A proteinase (2A^pro^) causes cleavage of the initiation factor eIF4GI and its paralog eIF4GII. This cleavage is augmented by viral activation of cellular proteinases that cleave eIF4G1 at multiple sites in the same region as 2A^pro^ [1,2,3,4,5,6]. eIF4GI is an Achilles heel in the cap-dependent translation mechanism since eIF4G is the central scaffolding protein that assembles the cap-binding protein complex eIF4F on the cap structure on mRNA and bridges the messenger ribonucleoprotein (mRNP) to the small ribosomal subunit through an interaction with eIF3 (Figure 1). The viral and cellular proteinases that cleave eIF4G separate the binding domain of eIF4G that interacts with the cap-binding protein eIF4E from the domain that binds eIF3, effectively preventing capped mRNAs from binding ribosomes *de novo* [7] (Figure 1).

Thus, cleavage of eIF4G strongly represses *de novo* cap-dependent translation initiation, but significant ribosome recycling, still a poorly understood process, seems to persist longer, enabling additional rounds of translation on established polysomes. In enterovirus-infected cells, viral cleavage of poly(A)-binding protein (PABP) by both 2A^pro^ and 3C proteinase (3C^pro^) blocks ribosome recycling, and cleavage of this second initiation factor is required to completely block cap-dependent translation [8]. Both the 2A^pro^ and 3C^pro^ cleavage sites on PABP are near one another and serve to remove the globular C-terminal protein interaction domain (PID) of PABP from the N-terminal RNA binding RRM motifs [9,10]. This PID domain interacts with both eIF4B in 5′ cap-binding complexes and ribosome release factor eRF3, which binds ribosomes at translation termination [11,12]. Viral proteinase cleavage of PABP is shared by caliciviruses, which also translate cap-independently, but do not induce cleavage of eIF4G during infection [13]. The final initiation factor cleaved by enterovirus proteinases, eIF5B, is cleaved by 3C^pro^ [14]. This cleavage plays no significant role in the initial host translation shutoff, but rather reshapes the translation apparatus so viral RNA can achieve eIF2-independent translation as will be discussed below.

## 2. Enteroviruses Promote then Selectively Block Cap-Independent Translation

Enteroviruses utilize cap-independent translation facilitated by the large viral internal ribosome entry site structure (IRES) located 120–620 nt downstream from the 5′ end. This structure has several stem loops and pseudoknots and serves to efficiently bind both canonical translation factors and other RNA binding proteins known as IRES-transactivating factors (ITAFs). ITAFs provide RNA-chaperone function, but may play other roles that help overcome translation restriction during cell stress or innate immune activation [15]. Recent work with *in vitro* reconstituted reactions indicates enterovirus IRESes minimally require several initiation factors; eIF2, eIF3, eIF4A, eIF4G, eIF4B, eIF1A, plus a single ITAF, poly(C) binding protein 2 (PCBP2) [16]. In addition, other ITAFs PTB, La autoantigen, UNR, and GARS have been shown to stimulate PV-IRES translation *in vivo* or *in vitro* [17,18,19,20,21,22]. Ribosome recruitment by the viral IRES still requires eIF4G, but only the C-terminal eIF3-binding domain and RNA-binding domains are required, not the N-terminal cleaved away by 2A^pro^ that contains the eIF4E binding site. The shutdown of cap-dependent translation from eIF4G cleavage has the effect of releasing ribosomes, thus reducing the competition for ribosomes by viral mRNA and promoting cap-independent translation on the viral IRES. Cleaved eIF4G promotes PV translation better than intact eIF4G [23], thus 2A^pro^ provides a host factor gain of function for the virus.

### 2.1. Viral Translation of Many Templates Is Only Transient

Plus strand RNA viruses all share a molecular biology problem in that the same infecting genome that is used to translate viral proteins and viral RNA must be converted into a template free of ribosomes (moving in the 5′–3′ direction) to enable RNA replication by viral RNA polymerase (that moves in the 3′–5′ direction). Thus, the virus at some point must also carefully block its own translation. For enteroviruses, this task is carried out by 3C^pro^. In a manner analogous to the shutoff of cap-dependent translation by 2A^pro^ cleavage of eIF4G, 3C^pro^ cleaves three ITAFs that support IRES translation, PCBP2, PTB and La [24,25,26]. These cleavages occur mid-phase in infection over a somewhat protracted phase. This serves to limit or restrict de novo translation on viral RNA since PCBP2 and likely the other ITAFs function in ribosome recruitment together with eIF4G, eIF4A and eIF4B) [16]. However ribosomes on existing viral polysomes can recycle similar to those on host mRNA polysomes. Thus, cleavage of PABP, which occurs more slowly, has the same effect of restricting recycling and promoting template clearance [27]. The result is a pool of viral RNA templates that are cleared of ribosomes and free to initiate RNA replication.

### 2.2. Viral Control of Innate Immune Factor Translation

Very little specific information is available concerning translation requirements of the mRNAs of most interferon stimulated genes (ISGs) and antiviral host factors. Some reports indicate that interferon (IFN) activated Jak-Stat signaling causes upregulation of mTOR and Mnk1 activity, thus promoting translation of a range of ISGs [28]. These changes promote cap-dependent translation in general, indicating that translation of many ISG mRNAs is cap-dependent, as predicted. This provides enteroviruses a huge advantage as expression of many innate factors will be severely inhibited after eIF4GI and PABP are cleaved during infection. From the standpoint of the cell, it makes evolutionary sense to have some adaptive host-defense genes that are translated by cap-independent IRES-mediated translation mechanisms that can resist the general shutoff of translation exerted by eIF4GI cleavage. Whether any innate immune factors translate by IRES-dependent or other stress-activated mechanisms is not known. However, recent evidence indicates one ISG fights back to hinder viral control of translation directly. ISG15 can conjugate to CVB3 2A^pro^, leading to restricted cleavage of eIF4GI [29] and presumably other host targets.

## 3. Enteroviruses Bypass Translation Inhibition Induced by Stress Responses

Infection by most viruses activates phosphorylation of the critical initiation factor eIF2. This factor lies at the center of numerous pathways linking stress-sensing to translation control. There are four eIF2 kinases, PKR (protein kinase R), PERK (double-stranded RNA-activated ER kinase), HRI (heme-regulated inhibitor), and GCN2 (general control nonderepressing 2). All function to repress translation of transcripts using AUG initiator codons (as nearly all do) by phosphorylating the alpha subunit of eIF2 on serine 51. This blocks eIF2-GDP recycling that is required to continually produce the eIF2–GTP–Met-tRNAiMet ternary complex which must bind 40S ribosomes to allow scanning of mRNAs for AUG start codons. Indeed, PKR, through its ability to bind viral dsRNA and self-activate, is also a key viral sensor and initiator of IFN and other innate immune responses. Many viruses have evolved mechanisms to counter PKR and IFN activation, including enteroviruses. PV is relatively resistant to type 1 interferon treatment *in vitro* and cleaves several interferon response activators such as RIG-I and MDA5 [30,31,32]. PV also causes partial degradation of PKR to limit stress responses and innate immunity [33], though the precise mechanism for this has not been reported.

Surprisingly, some viral IRES elements that use AUG initiation codons still promote translation after extensive eIF2α phosphorylation has occurred; termed eIF2-independent translation. These viruses include poliovirus, Hepatitis C virus, classical swine fever virus (CSFV) and also the cellular Src IRES [34,35,36,37,38]. This indicates that alternative protein complexes can somehow substitute for the normal eIF2-ternary complex to bind the initiator tRNA to ribosomes. In the case of CSFV, an eIF5B/eIF3 complex could substitute for eIF2/eIF3 function in the formation of 48S ribosome complexes with IRES-mRNA. For poliovirus, the gain in function was linked to cleavage of eIF5B by 3C^pro^ since the viral IRES by itself could not translate under conditions of high eIF2α phosphorylation. Instead, a cleavage fragment of eIF5B rescues IRES translation under stress conditions [38]. eIF5B is the eukaryotic homologue of archeal and prokaryotic initiation factor 2 (IF2), and like IF2, it is required for ribosomal subunit joining during translation initiation [39]. However, IF2 also performs the eIF2-like task of binding initiator tRNA to ribosomes in bacteria. The 3C^pro^ cleavage site in eIF5B was mapped upstream of the conserved C-terminal domain that is homologous to IF2 [14]. The N-terminal regulatory domain is removed by PV 3C^pro^ cleavage, essentially producing a protein similar to IF2, perhaps gaining more than a rudimentary tRNA-delivery function in the process [40]. In this way, PV generates a eukaryotic version of bacterial IF2, with a gain of function that promotes viral cap-independent translation long after eIF2α phosphorylation has shut down all other translation [38,40].

## 4. Enterovirus Control of Stress Granules

### 4.1. Stress Granules and Processing Bodies

Translation inhibition of variable degrees and duration is among the most common cellular responses to stress conditions. Cytoplasmic RNA granules are compartments for temporary storage of translationally-silenced transcripts, and are regulated together with upstream translational silencing mechanisms. The two main types of cytoplasmic RNA granules found in somatic cells are stress granules (SGs) and processing bodies (P-bodies, PBs). RNA granules are very dynamic and rapidly exchange components with the surrounding cytoplasm and can quickly appear and disappear.

Stress granules are condensates of stalled translation initiation complexes. They are not commonly found in actively growing cells, but form quickly in response to various types of environmental stress. Typically stress will activate one or more of the eIF2α kinases (PKR, HRI, GCN2, PERK), promote translation inhibition through eIF2α phosphorylation, and lead to accumulation of stalled translation initiation complexes. These initiation complexes are then quickly transported on microtubules and condense into SGs. Other pathways exist that promote SG formation without upstream eIF2α-phosphorylation, such as inhibition of eIF4A helicase function, cleavage of eIF4GI or other mechanisms [1,3,6,41,42].

Thus, SGs contain complex collections of mRNPs, including much of the proteome associated with cytosolic mRNAs such as translation initiation factors, 40S ribosomes, and regulatory mRNA-binding proteins. Many of these factors do not play important roles in SG functions. However there are a few RNA-binding proteins that play critical roles in SG assembly and disassembly. Such SG-nucleating factors include RasGAP-SH3 domain binding protein 1 and 2 (G3BP1 and G3BP2), T-cell-restricted intracellular antigen 1(Tia1), caprin1, Fused in sarcoma (FUS) and TDP-43, among others (Figure 1) [43,44,45,46]. Overall, the formation of SGs is seen as protective, enabling the cell to temporarily store a large variety of transcripts as the biochemical machinery reprograms, and promotes expression of transcripts that are required to overcome the stress. The stored transcripts can readily reenter the translation apparatus as the stress is overcome and recovery ensues.

Recently new insights have been gained in understanding how SGs form, thus providing new insights into how enteroviruses antagonize them. SG assembly requires multiple steps, including mRNP transport on microtubules, post-translational modification (PTM) of many mRNP proteins via phosphorylation, acetylation, methylation and O-linked *N*-acetylglucosamine, and ultimately requires protein-protein phase condensation functions of key nucleating proteins [47,48,49,50,51,52,53,54,55]. PTMs are thought to facilitate or trigger the altered protein-protein interactions that drive cytoplasmic condensate formation and provide interfaces with cellular signaling networks. For SGs, PTMs occur on a set of key proteins that play nucleating roles in SG formation, owing to the presence of aggregation or prion-like low amino acid complexity motifs in their sequences [56]. Importantly, RNA granules are not inert aggregates, rather foci of liquid phase condensates that display physical behaviors similar to liquid droplets. The distinction is key to how SG maintain high dynamic exchange rates of components with the surrounding cytoplasm yet retain a high packing density of mRNP components. SGs are not homogenous in their makeup or density. Recent work proposes SGs are nucleated in two stages from numerous small cores containing highly condensed mRNPs formed with nucleating proteins such as G3BP1, surrounded by highly dynamic, less dense constituents [57,58].

### 4.2. Stress Granules and Enteroviruses Have an Antagonistic Relationship

As with most viruses, enteroviruses assertively regulate many aspects of host gene expression to create conditions that promote the most efficient virus replication. This results in virus infection initiating multiple types of stresses on cells, including host translation shutoff discussed above. These stresses create SGs and increase the size and number of P-bodies, which both function as extensions of translation control mechanisms. Thus it makes sense that viruses have evolved to regulate these bodies to maintain maximum efficiency in translation of viral gene products, to avoid inclusion of viral mRNA as silenced transcripts within SGs and PBs and to prevent sequestration of translation components within SGs. In fact, enteroviruses antagonize RNA granules strongly, and this type of antagonistic relationship with RNA granules is common in other mammalian virus families (see reviews [59,60]). Whereas many viruses coopt key RNA granule components to neutralize their function, enteroviruses poliovirus (PV) and coxsackievirus B3 (CVB3) destroy RNA granule components through cleavage, which is discussed below. This aspect makes enteroviruses useful probes to study functions of RNA granule components.

### 4.3. Formation of SGs Is Driven by 2A^pro^ Cleavage of eIF4G and eIF2a Phosphorylation

As mentioned above, enterovirus infection results in rapid host translation shutoff through cleavage of eIF4G1, and PABP. This will produce thousands of idle, stalled host mRNAs, thus the cell will produce SG to compartmentalize and store the accumulating transcripts. So it is expected that enterovirus infection induces formation of SGs. In fact the first report testing the relationship of PV to SGs reported induction of SG in cells by 3 hpi, which was linked to eIF4G cleavage [6], and is thus linked to production of 2A^pro^ early in infection. The authors did not investigate the fate of SGs in the remainder of the PV infection cycle, thus the full story of SGs regulation remained undiscovered. The prediction that 2A^pro^ induces SG formation was confirmed more recently by expression of individual viral PV or CVB3 proteinases in cells [61,62]. Additionally, virus infection surely activates other types of stress that are sensed by eIF2 kinases, all of which are canonical activators of SG formation. As mentioned above, enterovirus infection is well known to activate PKR and drive accumulation of phospho-eIF2α, even though the virus prevents eIF2-phosphorylation from getting out of check until late in infection [33,38,63]. Thus, SG assembly is also promoted early in infection through depletion of met-tRNA ternary complexes. Because mechanisms that drive SG formation also occur simultaneously with other viral mechanisms that inhibit SG formation (discussed below), the SG formation actually observed in PV or CVB3-infected cells is transient and quite variable. Not all infected cells are observed with SGs (ranging from 15%–80% of cells) and their presence is dependent on time during the infection cycle and cell type [64,65].

After the initial phase of PV infection when SGs begin to form, they then disappear and are generally absent by 4 hpi through the remainder of the infectious cycle. PV produces an inhibitory activity that blocks the stress granule response that mobilizes in response to exogenous stressors such as arsenite, heat shock, and ER stress. This SG-inhibitory activity develops by 3 hpi and is the product of viral gene expression [64]. The pattern of transient SG induction followed by disruption, was later confirmed to occur in CVB3 and EV71 infected cells as expected [62,65].

### 4.4. Viral Cleavage of SG-Nucleating Protein G3BP1

How do enteroviruses block SG assembly? The process of RNA granule formation is complex and involves many steps and components, thus enteroviruses may be expected to control SGs through multiple channels/mechanisms. However, viruses with limited gene products often target the most important facets of a pathway. PV infection results in cleavage of the stress granule-nucleating protein G3BP1, but not two other important SG nucleating proteins Tia1 and TIAR [64]. G3BP1 is cleaved by PV 3C^pro^, which separates the major protein-interaction motifs (NTF2-acidic-PxxP) from the RNA-binding RRM portion of the protein [64]. Later work confirmed that infection with CVB3 or EV71 also results in G3BP1 cleavage and disassembly of SGs, with similar kinetics [62,65]. Further, a more distantly related cardiovirus, encephalomyocarditis virus (EMCV) also cleaves G3BP1 during infection of human cells [66]. All four viruses cleave G3BP1 at the same site, which is a canonical 3C^pro^ recognition site [64]. This evolutionary conservation attests to the importance of G3BP1 in SG assembly mechanism, though other critical functions of G3BP1 may be uncovered in the future. Although enterovirus proteinases are known to cleave several other host RNA-binding proteins such as ITAFs and PABP, it is unclear or unlikely that some of these are involved in SG dynamics, despite the fact that they have multiple roles in RNA biology. We have shown that cleavage of eIF4G does not interfere with SG assembly [67].

With multiple events and steps required to form SGs, can cleavage of G3BP1 play a dominant role in SG dynamics? G3BP1 contains five conserved domains and has a homolog G2BP2 that is nearly identical in the N-terminal NTF2 domain. Recent reports indicate G3BP2 also helps nucleate SG formation, and both G3BP1 and G3BP2 homodimerize and can form heterodimers [41,45,49,68]. However, siRNA depletion of G3BP1 only partly suppresses arsenite-induced SGs. This, together with other observations suggest G3BP1 and G3BP2 exhibit duplicity of function [56,64,69]. However our more recent work with Cas9/Crispr knockout cells indicates total G3BP1 ablation can severely block SG formation in response to arsenite in certain cell backgrounds [70]. Furthermore, G3BP2 is not cleaved by 3C^pro^ during infection, as the 3C^pro^ cleavage recognition site with a scissile glutamate-glycine bond at residue Q326 is not conserved in G3BP2 [61,64]. Expression of mutated cleavage-resistant G3BP1 in cells can preserve or restore SG formation during infection. Thus, cleavage of only G3BP1 by PV is associated with complete loss of SG formation, and the function of G3BP1 would appear to dominate over G3BP2 in SG assembly.

Why is the impact of G3BP1 cleavage on SGs so high? Despite much progress, the mechanistic role of G3BP1 in SG formation is still poorly understood. G3BP1 contains intrinsically disordered regions of low amino acid complexity (acidic, PxxP, RGG) that are implicated in condensation functions thought to nucleate RNA granules [58,71]. However, this proposed condensation function is similar to aggregation functions of the prion-like domains of Tia1 and TIAR, and at least 20 other proteins proposed to be involved in RNA granule nucleation. It is unclear how G3BP1 could be more important than these other RNA-binding factors with seemingly similar and overlapping functions. One possibility is that G3BP1 orchestrates higher-order complexes of factors required for SG formation. For instance, interaction of G3BP1 in complexes with itself, with caprin1 and TDP-43 are all important in SG assembly [43,46]. G3BP1 may function in the first step of a two-step model of SG assembly [57,58] by providing a SG-seed or primary protein condensate that then recruits other complexes.

Another possibility is that G3BP1 recruits a larger fraction of the cell mRNPs than other SG-nucleating proteins. G3BP1 binds mRNA through its RRM domain, however, the number of binding targets is unknown, but may be numerous, and includes c-myc and tau mRNAs [72,73,74]. Unfortunately, no RNAseq data are available to determine to what extent G3BP1, G3BP2, Tia1, TDP-43 or other SG nucleating proteins are bound to unique or overlapping sets of mRNAs in the cell.

A final hypothesis is that production of G3BP1 cleavage fragments may be more important than the loss of intact G3BP1 through the production of dominant negative inhibitors. The expression of certain G3BP truncations in cells revealed some degree of dominant negative activity [45]. The G3BP1 fragments generated by CVB3 3C^pro^ and the C-terminal RNA binding domain are inhibitors of SGs [65]. Further, deletion of the NTF2-like domain is sufficient produce a dominant negative factor [75]. Taken together, this may indicate that several interaction domains along the length of G3BP are needed for SG formation. Consistent with this interpretation, G3BP1 cleavage can unlink Tia1 aggregation from true SG formation. Late in infection after G3BP1 cleavage, small punctate aggregates of Tia1 remain in the cytoplasm, but these are not associated with ribosomal complexes and initiation factors that functionally define SGs [67].

### 4.5. Are There Other Virus-Targeted Host Factors That Regulate SGs?

Enterovirus proteinases cleave a growing list of host factors, thus, some known or unknown proteinase targets may also function in SG assembly or dynamics. Cleavage of eIF4G by 2A^pro^ was proposed to interfere with SG assembly, but actually does not since both *N*- and *C*-terminal cleavage fragments of eIF4GI can assemble into normal SGs in PV-infected cells under conditions where G3BP1 cleavage is blocked [64,67,76]. However, expression of 2A^pro^ in cells was able to partly repress arsenite-induce SGs, though not as stringently as expression of 3C^pro^, which cleaves G3BP1 [61]. Additional cellular targets of 2A^pro^ or 3C^pro^ that may play roles in SG formation have not been identified. Several ITAFs are cleaved by 3C^pro^ as mentioned above, and though PCBP2 and PTB colocalize weakly with SGs, a role for any ITAF in SG dynamics has not been proposed [77,78,79]. A well-known mRNA destabilizing factor, adenosine-uridine (AU)-rich element RNA binding factor 1 (AUF1), also destabilizes CVB3 and PV viral RNA [80,81] and is cleaved by PV 3C^pro^ in part to counteract this activity [82]. During CVB3 infection AUF1 is also weakly recruited to SGs, but this may not be relevant for SG dynamics and instead reflect incorporation of some mRNPs that contain AUF1 [83]. No role for AUF1 in SG assembly has been reported.

### 4.6. SG Inhibition by Other Picornaviruses

All human enteroviruses likely control RNA granules with the same mechanisms as PV and CVB3, since all encode both 2A^pro^ and 3C^pro^ with conserved cleavage specificities. However, many animal picornaviruses do not encode 2A^pro^. What can be learned from these viruses about SG regulation? Theiler’s murine encephalomyelitis virus (TMEV) represses SGs, but does not cleave G3BP1. [77]. The Q/G cleavage site in human G3BP1 is not conserved in murine G3BP1, however TMEV does not cleave human G3BP1 either, indicating evolutionary drift of protease specificities. In contrast, another cardiovirus, EMCV, blocks SGs in HeLa cells via cleavage of G3BP1 at the same site cleaved by PV 3C^pro^ [66]. It was not reported if EMCV can cleave murine G3BP1. Instead of G3BP1 cleavage, cardioviruses such as TMEV, Saffold virus and mengovirus all use the leader (L) protein, which is not a proteinase, to repress SG assembly. Mutation of the zinc finger motif of mengovirus L protein [84] or the Theilo domain of TMEV L protein [77] abrogated the ability to block SGs. The mechanism of how leader proteins repress SGs is unclear and could be indirect.

## 5. SGs as Signaling Platforms in Innate Immunity

Why would enteroviruses and so many other virus families evolve a myriad of strategies to block stress granule formation? Several reasons can be envisioned. First, a primary replicative strategy of enteroviruses is to take complete command of the translation apparatus to free ribosomes and other parts of the translation machinery to produce high levels of virus proteins quickly. Stress granules can been seen as counteracting this in two ways, they may sequester significant portions of small ribosomal subunits into compartments inaccessible to virus RNAs, and/or the virus RNAs themselves may be sequestered into SGs and PBs and thus translationally silenced. Both of these scenarios must be resisted by any effectively replicating enterovirus and it is known that PV RNA does not enter SGs during infection [76]. Lastly, emerging evidence suggests that SGs serve as signaling platforms that may regulate cell metabolism, survival and innate immune mechanisms. All these roles of SGs can be seen as potentially antiviral.

An intriguing hypothesis posits that SGs assemble signaling platforms to trigger aspects of innate immunity. If so, this would partly explain why so many viruses antagonize SGs during their replicative cycles. Viruses have co-evolved with their hosts for millennia, have always induced various types of stress during infection and so linkages between stress-sensing mechanisms and innate immunity are plausible. Much of the innate immune response requires recognition of pathogen associated molecular patterns (PAMPs) by pattern recognition receptors (PRRs) such as Toll-like receptors, RNA-recognition molecules (e.g., RIG-I, MDA5), *etc*. These specific interactions between PAMPs and PRRs trigger activation of the interferon system and nuclear factor κB (NF-κB)-associated innate immune signaling. Whereas PRRs rely on direct pathogen molecular interactions, an alternate innate immune activation system promoted by stress and stress granule assembly would not, extending defenses to agents where PAMP-PRR interaction may not occur. A stress-associated signal could synergize with PAMP-PRR signaling as well. Additionally, cell stress responses that lead to SG formation are associated with pro-survival mechanisms. SGs down-regulate apoptosis by including RACK1 or WDR62 into SG to repress c-Jun N-terminal kinase (JNK) signaling [85,86].

Several connections between innate immune factors and stress granules have been reported, yet for many, regulatory roles related to SGs have not been demonstrated. Many innate immune activators and downstream ISGs (PKR, LGP2, MDA-5, RIG-I, RNase L, OAS) can be found in SGs formed during influenza virus infection and depletion of G3BP1 or PKR reduces IFNβ mRNA production [87,88]. One way that SG formation may activate innate immunity is by concentrating key proteins, thereby increasing interactions between PRRs, their targets, and signaling molecules. SG assembly during paramyxovirus infection may promote interaction between RIG-I and MEX3C, which is a ubiquitin ligase that ubiquitinates RIG-I and aids activation of the IFN-β promoter [89]. Though concentration of innate immune factors in SGs seems a logical way to amplify signaling, this may not always be the case. Both MDA5 and RIG-I enter mengovirus-induced SGs, but MDA5 activation does not occur even though formation of SGs is associated with reduced mengovirus replication [84,90]. However, more recent work indicates RIG-I is activated by sensing uncapped paramyxovirus vRNA that is selectively captured in SGs [91].

PKR is a prototypical ISG and also serves as one of four canonical activators of SG through phosphorylation of its primary substrate eIF2. However, PKR is also an important signaling factor and can initiate innate immune transcriptional activity and activate inflammasomes in macrophages [92,93,94]. PKR also helps regulate JNK activation, and coordinates metabolic homeostasis with pathogen sensing [95]. PKR can be recruited to SGs induced by influenza virus infection or after its activation with dsRNA [88,96]. In either case it was not determined if any activation of PKR or other regulation occurs when PKR associates with SGs.

### 5.1. G3BP1 Is Antiviral

We have shown that SGs induced by G3BP1 overexpression are antiviral against multiple enteroviruses [64,75]. The granules induced by G3BP1 in the absence of virus infection can assemble OAS and RNase L into SGs similar to influenza virus-induced SGs [75]. Assembly of large G3BP1-induced SGs also causes PKR activation that results in downstream phosphorylation of eIF2α [41]. G3BP1 plays a key role in PKR activation as it directly interacts with unactivated PKR together with its cofactor Caprin1, and recruits PKR to SGs (Figure 1). PKR activation occurs during SG assembly and then it is released from the G3BP1 complex located within SGs to cycle back to the cytoplasm to interact with its substrates [69,75]. These findings elucidate a new PKR activation pathway that is dependent on G3BP1 incorporation into SGs, and show that SGs can operate as signaling platforms to activate innate immunity. G3BP1 may also activate ISGs via another mechanism. G3BP1, its homolog G3BP2 and Caprin1, are all RNA-binding proteins involved in translational activation of some ISGs in response to IFN. Interestingly, this includes increased translation of PKR mRNA [97].

So what is the impact of stress granule innate immune signaling on enteroviruses? Evolutionary pressure drove enterovirus proteinases to substrate specificities that cleave a remarkable set of key translation factors, but also critical innate immune signaling proteins, including MDA5, RIG-I, MAVS, IRF7 and TRIF [30,31,32,98,99,100]. To these we now can add G3BP1, whose cleavage simultaneously promotes translation and restricts innate immune activation, significantly promoting virus replication [75]. More work will be required to learn whether enteroviruses cleave or inactivate additional SG components that can be mobilized by inclusion in granules. It should also be pointed out that transcriptional activation of any innate response is directly antagonized by 2A^pro^ and 3C^pro^ through cleavage of TATA binding protein and restriction of Pol II function [101,102]. Further, as discussed above, translation of ISGs and innate immune cytokines is inherently cap-dependent, and will be severely blocked by the cleavage of eIF4G and PABP. Together, enteroviruses destroy an impressive array of host defense and gene expression mechanisms through the multiple targets of 2A^pro^ and 3C^pro^.

## 6. Enteroviruses Disperse P-bodies

**Figure 2:**
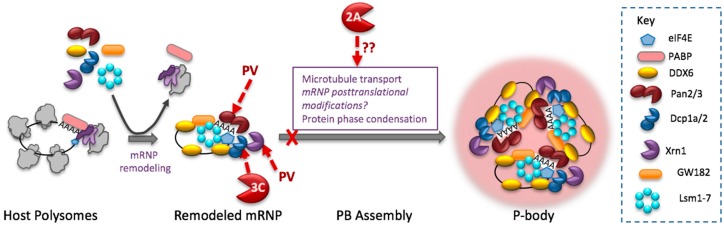
Enterovirus inhibition of P-bodies. Cartoon depicts mRNP remodeling required for assembly into PBs and cleavage of PB factors that destabilize mRNA. Pan 3 and Dcp1a play roles in PB formation. 2A^pro^ blocks PB formation through an unknown mechanism(s).

Processing bodies, which are constitutively present in cells, are quite different from SGs. The mRNP composition of PBs is distinct from SGs; PBs lack 40S ribosome subunits and translation factors but are enriched in RNA decay machinery such as Xrn1, decapping factors Dcp1a, Dcp2, EDC3,4 and poly(A) nucleases Pan2, Pan3, Ccr4, Not1 and the Lsm1 exosome (Figure 2) [103,104]. As a focus of RNA decay, PBs potentially represent a threat to enterovirus RNA. In particular, viral mRNA is at risk for 5′ end mediated degradation since it is not capped or protected by VPg like genomic RNA [105,106].

PBs are quickly dispersed in cells infected with PV or CVB3, and are virtually absent in cells by 4 hpi, near the time virus RNA synthesis is reaching peak levels [107]. The mechanisms of PB assembly are unknown, but hypothesized to involve the same principle of liquid phase changes mediated by interactions between a different set of RNA-binding proteins bearing low amino acid complexity domains, potentially decapping complex proteins [56,104]. Some specific proteins that may contribute to PB formation are Rck/p54 (also known as DDX6), Dcp1a, Xrn1, LSm14A, Dcp2, GW182 and EDC3 [47,104,108,109,110].

Interestingly, PV replication results in cleavage/degradation of two of these factors, Dcp1a and Xrn1 (Figure 2). PV 3C^pro^ cleaves Dcp1a, removing a *C*-terminal domain and phosphorylation site that mediates Dcp1a trimerization, though the exact cleavage site has not been mapped. In uninfected cells Dcp1a trimerization promotes decapping of mRNA and also mediates Dcp1a localization to P bodies [111,112]. Xrn1 is the major 5′-3′ exonuclease involved in mRNA decay. PV infection destablizes Xrn1, shortening its half-life by 3-fold, however, neither PV 2A^pro^ nor 3C^pro^ cleave Xrn1. Instead Xrn1 degradation stems from viral stimulation of proteasomal degradation [107].

PV infection also causes degradation of the poly(A) nuclease subunit Pan3 (Figure 2). Depletion of Pan3 inhibits mRNA deadenylation required for mRNPs to be included in PBs [113], thus Pan3 cleavage may directly downregulate PBs by restricting input of mRNP cargo. Pan3 cleavage may also benefit viral mRNA stability by preserving poly(A) tails lengths and restricting translation-associated poly(A) shortening that cellular mRNAs undergo [114,115].

A recent expression screen revealed that several polioviral proteins inhibit the formation of PBs in cells when expressed individually. These included 3C^pro^, which was expected since Dcp1a is a molecular target. However, 2A^pro^ also strongly inhibited PBs, whereas 3CD and 3Dpol only slightly reduced the number of PBs [61]. The mechanism by which 2A^pro^ inhibits PBs is unknown and 2A^pro^ does not cleave any host proteins currently thought to aid PB assembly, including EDC3, Xrn1, GW182 and Dcp2 [107]. In addition, 2A^pro^ and 3C^pro^ block different pathways that promote PB assembly. Oxidative stress promotes PB assembly, and application of this stressor could rescue PBs in cells where 2A^pro^ was expressed. In contrast, in cells expressing 3C^pro^, PBs could not be rescued by oxidative stress [61]. These data indicate that enteroviruses utilize several pathways simultaneously to disperse PBs in cells.

### 6.1. Can P-bodies Promote Innate Immunity?

Since P-bodies also form in response to stress it is possible that pathogen surveillance and innate immune signaling could be triggered through these RNA granules as well. Little is known with regard to PB mediated signaling but we have shown that crosstalk exists between one PB component and PKR that can block enterovirus infection. 3C^pro^ cleaves Dcp1a [107], which is a regulator of mRNA decapping. This is a surprising target for PV since enterovirus RNAs are never capped, and thus would not be predicted to play a role in viral RNA stability. Our studies revealed that expression of Dcp1a results in strong activation of PKR, eIF2α phosphorylation and restriction of replication [116]. Unlike G3BP1-induced PKR activation, PB formation was not required for Dcp1a-induced PKR activation, thus the constituents of PBs may be more important than the granules themselves in restricting virus replication [116]. Nonetheless, eIF2α phosphorylation induced by Dcp1a expression indicates that novel signaling crosstalk exists between RNA decay, translation regulation, and innate immunity.

## 7. Concluding Remarks

Enteroviruses have developed multiple strategies to evade host defenses and take over gene expression machinery in the host cell. Most of the known viral mechanisms involve the two viral proteinases, and study of protease targets has uncovered several key regulatory checkpoints in host gene expression. G3BP1 is a relatively new viral protease target and has emerged as a critical factor in SG assembly, and now also in innate immune activation. Future work with enteroviruses should address remaining questions and vigorously pursue unknown viral protease targets. How are P-bodies dispersed, and what is the mechanistic role of 2Apro in this? How exactly does G3BP1 scission destroy SG assembly? What is the mechanism of Dcp1a activation of PKR, what are the cofactors, and how else may the RNA decay pathway be linked to innate immune signaling? It will be fascinating to see progress in these areas and how new insights may be exploited to control virus infection and produce novel antiviral therapeutic agents. Finally, the emerging crosstalk between stress, innate immunity and inflammation suggests future novel strategies to control stress-triggers of autoimmune disease and other non-viral diseases may be possible.
